# Adherence to Mediterranean Diet in Croatia: Lessons Learned Today for a Brighter Tomorrow

**DOI:** 10.3390/nu14183725

**Published:** 2022-09-09

**Authors:** Marko Gerić, Katarina Matković, Goran Gajski, Ivana Rumbak, Paula Štancl, Rosa Karlić, Martina Bituh

**Affiliations:** 1 Institute for Medical Research and Occupational Health, Ksaverska Cesta 2, 10 000 Zagreb, Croatia; 2 Faculty of Food Technology and Biotechnology, University of Zagreb, 10 000 Zagreb, Croatia; 3 Faculty of Science, University of Zagreb, 10 000 Zagreb, Croatia

**Keywords:** Mediterranean diet, lifestyle, nutrition, health, dietary plan

## Abstract

Non-communicable diseases (NCD) and lifestyle, particularly diet, have a close relationship. Based on the recent statistics, Croatian men and women lead in European overweight lists, which implies pessimistic prognosis in terms of incidence and prevalence of NCDs in the future. One of the possible solutions to overcome weight problems is turn to traditional balanced and sustainable diets, such as the Mediterranean diet. In this study, we assessed adherence towards Mediterranean diet using a validated questionnaire in an online survey and associated adherence scores with several demographic and anthropometric data. Based on the results of a validated Mediterranean Diet Adherence Screener (N = 3326), we assessed the adherence score to be 7.6 ± 2.5. The score tended to depend on sex, residence, age, education, income, and body mass index (BMI); indeed, women, residents of a coastal part of the country, older volunteers, those possessing a higher education degree, those with higher income, and those with lower BMI were associated with higher scores. As income was one of the significant findings related to higher adherence scores, we developed a dietary plan complying with Mediterranean diet principles that, on average, costed less than the average traditional balanced diet menu. Taken together, this study brought new findings regarding target groups who need to be encouraged to make lifestyle changes, and highlighted the first steps on how to make them.

## 1. Introduction

Non-communicable diseases (NCDs) are a leading cause of death globally, accounting for roughly 75% of deaths in 2019. This trend is more pronounced for high-income and upper-middle-income countries, where nine of the top ten estimated leading causes of deaths are attributed to NCDs [1]. The latest statistics indicate that, in 2017, 56 million people died, of which 48% were cardiovascular (17.8 mil) and cancer (9.6 mil) patients [2]. Regarding the incidence and outcome of those diseases, lifestyle has been identified as one of the key drivers. Smoking, excess body mass, lower physical activity, and poor nutrition are the most important constituents of lifestyle contributing to overall morbidity [3,4,5]. During the past 2 years, the COVID-19 pandemic emerged, accounting for more than 5 million deaths so far, and excess mortality of more than 80 deaths per 100,000 compared to the 5-year average in several countries [6]. Although COVID-19 is a communicable disease, a similar association was found, where worse lifestyle and obesity as a comorbidity led to worse disease outcomes [7,8]. Based on these data, we can suggest that targeting lifestyle is key to better disease prevention.

According to EUROSTAT, in 2019, Croatian males and females led the negative lists of prevalence of overweight individuals in Europe [9]. Moreover, recent reports suggest that Croatia has one of the highest prevalence in European countries for childhood overweight and obesity, indicating negative projections for the future and an even higher burden for society and the healthcare system [10,11,12,13,14,15,16]. At the same time, according to Eurobarometer, food price is the most important aspect in terms of food purchase for Croatians and the third most important aspect for EU citizens, behind taste and food safety [17]. Such an attitude is more pronounced in low-income populations, which prefer calorie-dense and usually more processed foods [18].

On the other hand, these data are almost paradoxical considering that Croatia is a Mediterranean country with a long tradition of Mediterranean diet (MedD) and lifestyle. MedD has considerable beneficial effects towards weight control, cardio-vascular diseases (CVD), cancer, mental health, etc. [19,20,21,22,23]. It is characterized by high and frequent intake of olive oil, nuts, vegetables, and fruit, moderate intake of fish and seafood, and poultry, and low intake of red and processed meat, saturated fat, and sweets, accompanied by moderate red wine consumption enabling a balanced intake of energy, macro- and micronutrients, as well as phytochemicals important for human physiology [24,25,26]. Although it has beneficial properties, the trends of abandoning dietary patterns characteristic of MedD are being observed in many countries, including Croatia [27,28].

From the available literature, we could detect that the Croatian population has weight control issues that substantially increase disease risks, and that lifestyle and dietary changes might improve the current situation. However, there is no comprehensive screening of the population regarding MedD practicing habits; therefore, we aim to assess the dietary habits of the Croatian population with the focus on MedD dietary patterns, and relate them with demographic and anthropometric data. Moreover, we tailored a weekly plan that would comply with all the principles of MedD and be less expensive compared to a traditional dietary plan. We see this study as the first step in initiating stronger activities related to raising awareness of MedD benefits and health issues in Croatia and ‘Western’ societies, as well as for development of strategies and activities related to improvements in health and quality of life.

## 2. Materials and Methods

### 2.1. Ethics

The Ethics committee of the Institute for Medical Research and Occupational Health approved the study, assuring the high principles of ethics in scientific research, privacy of results, and anonymity of participants.

### 2.2. Mediterranean Diet Adherence Questionnaire, Its Refinement, and Adaptation for Online Use

For the assessment of MedD adherence, we used a validated 14-point Mediterranean Diet Adherence Screener developed by Schröder et al. [29]. The questionnaire covers intake of olive oil, vegetables, fruit, red meat, fat, beverages, wine, legumes, seafood, pastry, nuts, and dishes based on tomato and olive oil, as well as preferences of olive oil, and white and red meat intake. Each question was scored with 0 or 1 based on participants’ answers. The range of possible MedD score was 0 to 14.

**Figure 1:**
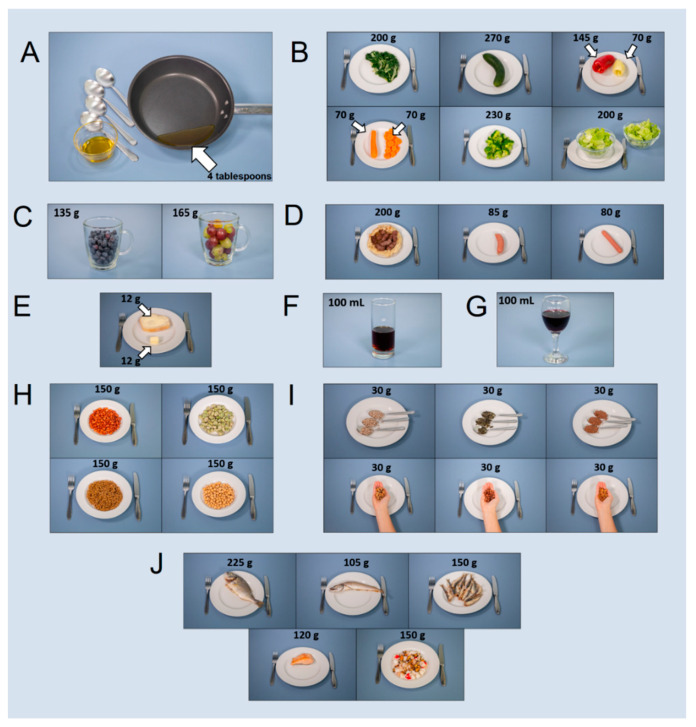
Photographs of representative food items based on EFSA’s national food consumption data and mass/volume for questions in the Mediterranean diet adherence questionnaire: (**A**) olive oil, (**B**) vegetables: chard, cucumber, pepper, carrot, broccoli, and lettuce, (**C**) fruit: blueberry and grapes, (**D**) meat: grilled minced meat “ćevapčići”, Kranj sausage, and Frankfurt sausage, (**E**) butter, (**F**) sweetened beverage, (**G**) red wine, (**H**) legumes: beans, broad beans, lentils, and chickpeas, (**I**) seeds and nuts: sunflower seeds, pumpkin seeds, flax seeds, walnuts, hazelnuts, and almonds, (**J**) fish and seafood: gilt-head bream, hake, European pilchard, salmon fillet, and mixed seafood.

The whole questionnaire was refined and adapted for online assessment using the Surveyplanet app using Pro features. The questionnaire refinement included food photographs to help the participants estimate portion size. Most of the questions were accompanied with a few photographs of representative food items (Figure 1). Representative food items and portion size were defined according to the national food consumption data [30]. The food photos were taken by a professional photographer using a digital camera (Canon) mounted on a tripod with constant lightning and angle according to guidelines by Ocké et al. [31].

### 2.3. Study Population

To recruit participants, the invitation link to participate in a study was sent using several social media platforms, non-governmental organizations’ mailing lists, and posters and flyers with a QR code put up in public places, initiating non-probability snowball sampling. Before taking part in a study, participants read the informed consent and agreed to participate. The study was limited to one response per device, disabling multiple answers from the same person, including those who declined to participate in a study (N = 16). In addition to questions to assess MedD score, we collected basic anthropometric and socio-demographic data, as well as personal perception of adherence to MedD. The survey was active for a whole year (March 2020–March 2021).

To assure the reliability of the results, we approached participants who gave their consent and performed a repeatability study with the same set of questions. The repeatability study was performed at least 3 weeks from taking part in the original survey.

### 2.4. Price Assessment for MedD

To determine average daily MedD cost, we used two different approaches. The first approach was to compare the prices of a MedD dietary pattern and a general healthy dietary pattern. Criteria for both dietary patterns were taken from the Dietary Guidelines for Americans 2020–2025 [32]. Diet plans for both dietary patterns were set to 2000 kcal per day. The following criteria were met for MedD: 184 g (6.5 oz) of protein foods, 27 g of oils, 170 g (6 oz) of grains, 2.5 cups of fruit and vegetables, and 2 cups of dairy per day. The healthy diet plan differed from MedD in the amount of fruit (2 cups vs. 2.5 cups), and dairy (3 cups vs. 2 cups). Additionally, the healthy diet plan differed in the lower total amount of protein food (5.5 oz vs. 6.5 oz in MedD), but also in the lower amount of protein food subgroup–seafood (8 oz vs. 15 oz in MedD per week). Average price per food group was estimated using data on the recommended intake according to the Dietary Guidelines (weight and frequency) and prices of selected locally consumed food. Locally consumed foods were selected from the Mediterranean food list for each food category [33]. For each food item, price per gram was multiplied by the grams that represent the portion (cups or ounce) of that food item. Price per portion of all food items in a certain food group was calculated, and consequently an average price for that food group was determined. Finally, the average price of a food group was multiplied by the recommended number of portions. We used prices from a nation-wide retailer providing the same food items and equal prices.

The second approach was to create 7-day MedD menus using the above-mentioned criteria, as well as the recommendation of weekly amounts for vegetable subgroups (dark-green vegetables; red and orange vegetables; beans, peas and lentil; starchy vegetables and others) and protein foods subgroups (meat, poultry, eggs; seafood and nuts, seeds, soy products). The price for a 7-day MedD was averaged by the price for each day.

### 2.5. Statistical Analysis

Statistical evaluation was done using R statistical programming language version 4.1.0. Missing values from the data were imputed using the R package mice [34]. Basic statistical parameters were obtained using descriptive statistics. Basic statistical parameters were calculated by applying the basic statistic method and frequency tables. The correlation of various features was done by calculating Spearman’s correlation. MedD scores were divided into quantiles and labelled Low (0–4), Intermediate (5–9), and High (10–14). The normality of continuous variables was checked using the Shapiro–Wilks W test. Afterwards, the difference between features between two quantiles was assessed by a Wilcoxon rank sum test, and among multiple groups by the Kruskal-Wallis test. A two-proportion Z-test was used to assess the difference in proportions within different features in each MedD score quantile. The influence of features on MedD scores was tested by the ordinal logistic regression model. The Wilcoxon signed-rank test was used to test the differences in MedD score before and after the initial study. Benjamini–Hochberg correction was applied to *p*-values, and the significance level in all of the tests was set to *p* < 0.05.

## 3. Results

The total study population consisted of 3342 volunteers, of which 3326 agreed to participate in the study (Croatian population in 2021 is 3.88 mil); 77.3% of volunteers were female, 22.7% male, while the average age was 37.4 ± 12 years. In total, 66.7% of volunteers possessed a university or higher degree, 33.3% had other educational levels. Half of the volunteers were never smokers, 23.2% were ex-smokers, and 26.8% were current smokers. The majority of participants were from the continental area, around 73%, while the rest were from the coastal area. The average MedD score for all participants was 7.6 ± 2.5.

**Figure 2:**
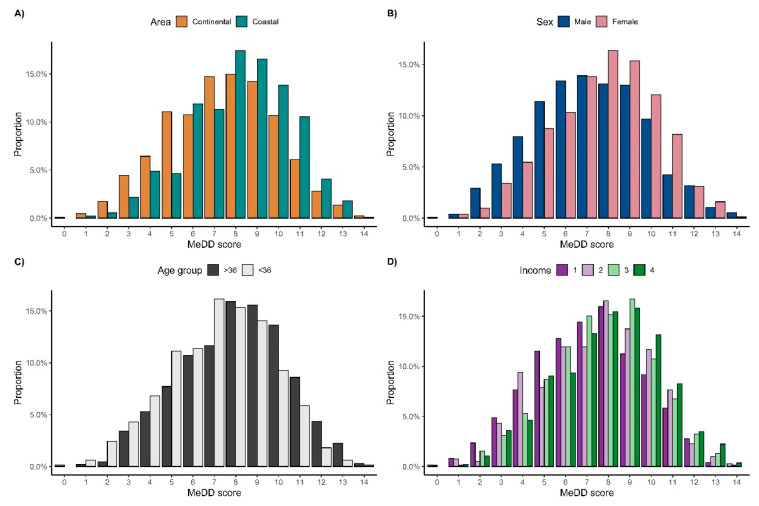
Distribution of volunteers based on a Mediterranean diet score according to: (**A**) area of residence—either continental or coastal part of Croatia; (**B**) sex—male or female; (**C**) age—those younger or older than median of 36 y; (**D**) income—where 1–4 represent income quartiles based on the National Bureau of Statistics data.

The average MedD was significantly higher in participants from the coastal area, at 8.2 ± 2.3, compared to those from the continental area, at 7.4 ± 2.5, (Wilcoxon rank-sum test, *p* < 0.001). Higher proportions of participants from the coastal area were detected in higher MedD scores in comparison to the proportion of participants from the continental area (Figure 2A), well above the calculated average MedD score. Women also had higher MedD scores when compared to men (Figure 2B). The same trend was observed for participants above the median of 36 years of age (Figure 2C). Higher proportions of participants whose income was in the upper two income quartiles, based on the National Bureau of Statistics data, had a MedD score above the median, while those whose income was in the lower two income quartiles tended to have a MedD score below the median (Figure 2D).

**Figure 3:**
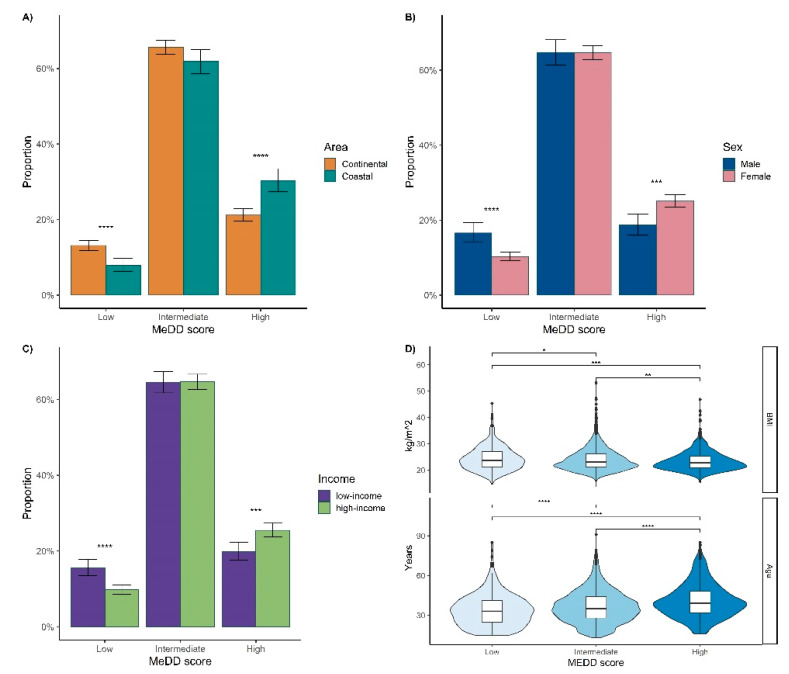
The distribution of volunteers when grouped into low (0–4), intermediate (5–9), and high (10–14) Mediterranean diet score groups according to: (**A**) area of residence—either continental or coastal part of Croatia; (**B**) sex—male or female; (**C**) income—where low or high-income was based on the National Bureau of statistics median data (**D**) body mass index (BMI) (kg/m^2^) and age. *: *p* < 0.05, **: *p* ≤ 0.01, ***: *p* ≤ 0.001, ****: *p* ≤ 0.0001.

Division of MedD scores into low (0–4), intermediate (5–9), and high (10–14) groups further validated these findings. There is a significant difference according to the different areas of residence, sex, and income in the high and low MedD score groups (two-proportion Z-test, *p* < 0.05) (Figure 3A–C). On the other hand, for the intermediate MedD score groups, there was no significant difference according to any of the analyzed features. Age and body mass index (BMI) values were significantly different between three groups of MedD scores (Kruskal-Wallis test, *p* < 0.001) (Figure 3D). Participants from the low MedD group were significantly younger compared to the intermediate and high MedD score group (one-sided Wilcoxon rank-sum test, *p* < 0.05). On the contrary, the BMI values were the highest in the low MedD group compared to the other MedD score groups (one-sided Wilcoxon rank-sum test, *p* < 0.05).

**Figure 4:**
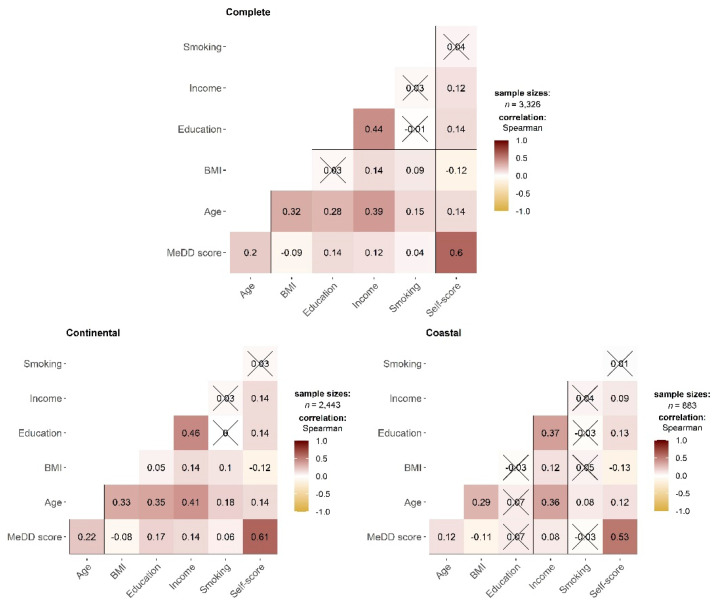
Spearman’s correlation plot for different parameters and Mediterranean diet score for the entire study group, as well as for separately presented volunteers from the continental and coastal parts of Croatia. Checked boxes indicate that correlation was not statistically significant (*p* < 0.05).

Spearman’s correlation analysis showed a significant correlation between MedD score and age, BMI, level of education, income, smoking habits, and self-score in the complete cohort study (Figure 4). When analyzing the cohort separately based on the residency area, only education and smoking habit did not significantly correlate with the MedD score in participants from coastal parts of Croatia. The strongest significant and the most positive correlation is between self-score and MedD score (Spearman’s correlation = 0.6), followed by correlation of income and education (Spearman’s correlation = 0.44) in the complete cohorts. A similar trend was observed when separating the study group according to the residential area.

**Figure 5:**
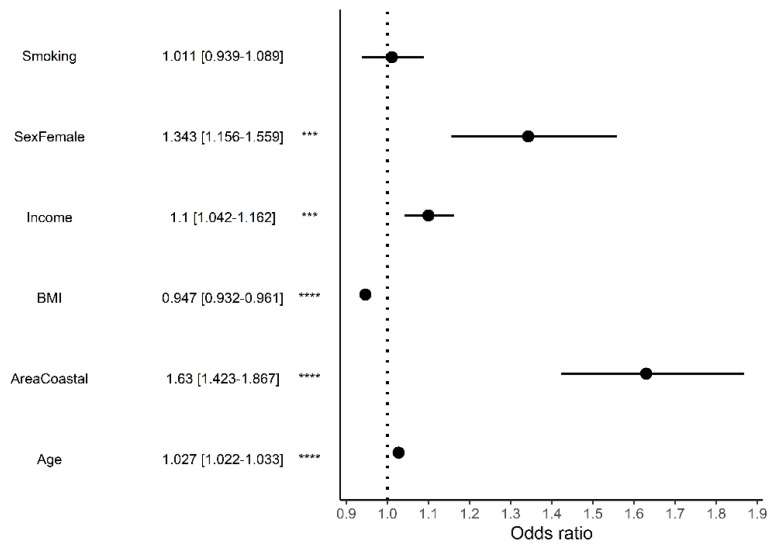
Ordinal logistic regression odds ratio and 95% confidence intervals for each parameter contributing to the Mediterranean diet score. ***: *p* ≤ 0.001, ****: *p* ≤ 0.0001.

Due to the high correlation of these features, self-score and education were excluded from ordinal logistic regression for analysing the influence of different features on the MedD score. The most significant features with the highest odds ratio detected with ordinal logistic regression are coastal areas (1.63, 95% CI [1.423–1.867]) and being female (1.343, 95% CI [1.156–1.559]) (Figure 5). Only the smoking status did not have a significant influence on the MedD score.

**Figure 6:**
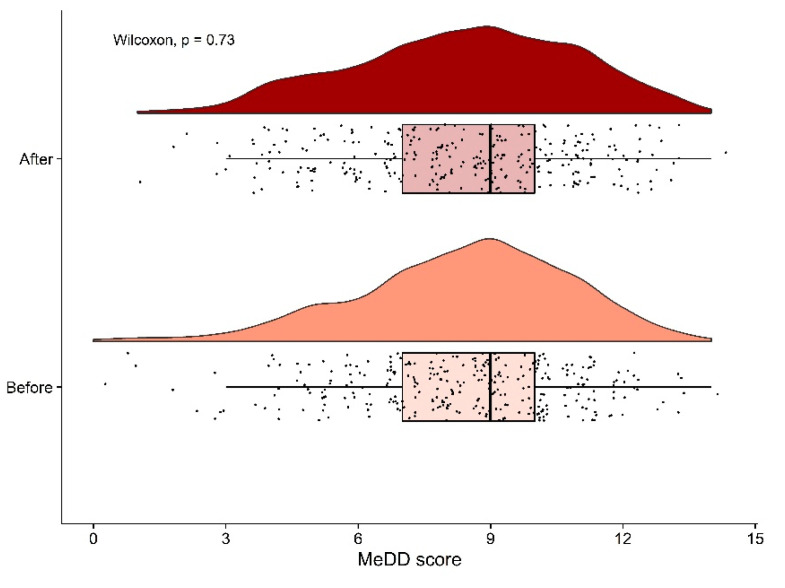
The results of the repeatability study with box plots and the distribution of Mediterranean diet score before and after the initial survey. No statistical differences were observed (Wilcoxon signed-rank test, *p* = 0.73).

Participants who gave consent and their contact details (605 out of 3326) were asked to answer the same set of questions to assess the reliability of the results. We received 335 replies; of those, 29 people changed their dietary habits so they were excluded from the repeatability study, leaving us with a 50.6% response rate. No significant difference was detected between the MedD score obtained before and after the initial analysis (Wilcoxon signed-rank test, *p* = 0.73) (Figure 6).

**Figure 7:**
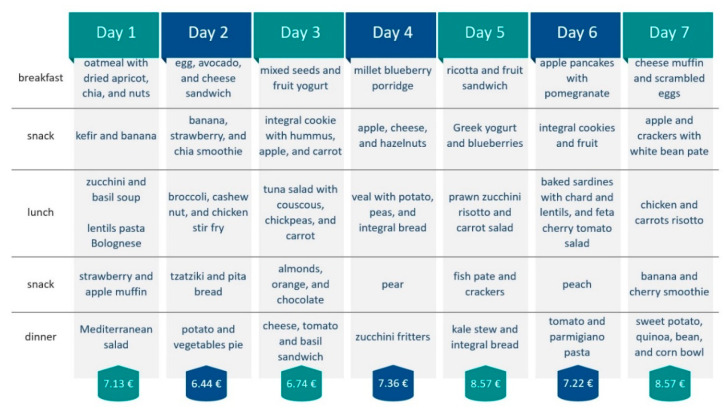
Tailored 2000 kcal, 7-day dietary plan based on Mediterranean diet principles and its cost per day.

In order to provide a weekly MedD dietary plan based on the calculation described in Section 2.3, and to target the low-income group as seen in Figure 2D, Figure 3C and Figure 5, we found that an average daily MedD plan per person is on average cheaper (EUR 9.47) compared to a traditional balanced plan (EUR 11.25). Moreover, in Figure 7, we managed to tailor a weekly MedD plan that would require an average of EUR 6.98 per day by choosing more affordable food items, thus potentiating its affordability.

## 4. Discussion

Weight control is an obvious global epidemiological problem for many countries, including Croatia [9,35,36]. Energy-dense and nutrient-poor food items are characteristic of the Western diet, which is a key factor, along with a sedentary lifestyle, for obesity development. Studies found that obese individuals, among others, suffer from induced metabolic stress, which alters the gut microbiota, while a pro-inflammatory environment stimulates senescence. Therefore, being overweight or obese indicates a significant risk for development of many other non-communicable diseases, such as CVD, cancer, and diabetes [37,38,39]. Mediterranean diet, over the last few decades, has come into the spotlight due to having multiple beneficial properties to humans, including improvements in gut microbiota, vascular ageing, inflammatory and other health-related biomarkers, preventing certain cancer types, reduction in overall mortality, and combating CVD, thus potentially leading to substantial annual savings [19,21,40,41,42,43,44,45,46,47,48,49,50]. In addition to many health and nutrition benefits, the Mediterranean diet, as a mainly plant-based diet, contributes to lower greenhouse emissions and a smaller water footprint, while promoting biodiversity. From 2010, UNESCO adopted MedD as an Intangible Cultural Heritage of Humanity, thus promoting the Mediterranean lifestyle as an invaluable socio-cultural achievement characterized by socialization, moderation, physical activity, and adequate rest. Finally, MedD contributes to positive local economic returns by protecting traditional production procedures and reducing the dependence on food import of local communities [20,25].

To enable rapid assessment of MedD within the population, we adapted a Mediterranean diet adherence questionnaire for online reporting. Since one of the major limitations in food consumption self-reporting is the over/underestimation of food portions, we accompanied most of the questions with photographs of representative food items and their portion sizes. Online surveys have the ability to reach many people in a short period of time, with flexibility, and also low administration costs [51]; they proved to be quite useful, particularly in the period of heavy restrictions of mobility and accessibility due to the COVID-19 pandemic. Moreover, based on our results, the online survey proved to be reliable in assessing MedD score. On the other hand, females, people with a higher education degree, and those with high-income are more prone to participate in such a survey [52], which was also the case in our study.

After analysis of the results, we found that adherence to MedD is higher in people residing in coastal parts of Croatia, in women, in people older than 36, and in people with higher income. The score significantly correlated with education level and was inversely correlated with BMI. Such trends are in accordance with similar studies [28,47,53,54,55,56,57,58,59,60,61,62,63,64,65,66,67]. Overall, the score of 7.6 ± 2.5 out of 14 could be considered as a medium MedD adherence level, which was in accordance with other Mediterranean [53,54,55,56,57] and non-Mediterranean countries [58,68,69]; however, certain specific populations might show poor to average adherence [70,71]. Still, we have to interpret results with the limitation that this study group was not representative for Croatia, since it comprised of > 75% women and > 50% of people with a university degree. Taking these factors into account, together with the results of the study by Pfeifer et al. [55] where it was concluded that Croatians tended to increase MedD adherence during pandemics, we could speculate that the actual MedD adherence score for Croatia is in fact lower than 7.6.

Concerning trends indicating abandoning MedD patterns have shown a significant decrease in Mediterranean adequacy index (MAI) over the last 50 years. On top of that, of top ten countries with biggest MAI decrease, nine were Mediterranean countries and Japan [27]. It is interesting to point out Japan, where the Okinawan diet is also characterized to be one of the drivers for longevity; however, the trends of westernization led to dietary changes, followed by a sharp increase in obesity and metabolic syndrome [72,73,74]. On the other hand, more optimistic trends are seen in the last decade, indicating the stabilization of MAI values which in some countries rose, while MedD adherence remained more or less the same [27,57,63].

When inspecting certain food items, we observed that 85.5% of volunteers did not meet the required red wine consumption, 69.9% of volunteers did not meet the required fish and seafood consumption, and 59.5% of volunteers did not meet the required olive oil intake. On the other hand, 83% of volunteers met the criteria for sweetened beverages intake, and 76.4% met the criteria for the intake of fat. These data are in line with the most recent paper investigating dietary preferences in the Croatian population [55]. In terms of health benefits, red wine is abundant in different polyphenols with potentially beneficial properties, while its consumption is associated with the “French paradox” and the reduction of coronary heart disease [75]. Fish and seafood is regarded as a source of high-quality protein and fat, particularly omega-3 fatty acids that were shown to reduce serum triglycerides and the risk of coronary heart disease [76]. Olive oil is highly valued because of its fatty acid and polyphenolic composition, which are key in risk reductions of CVD [77,78]. Based on the results of self-awareness towards complying with MedD patterns, the volunteers were quite accurate in their perception (2.96 ± 0.98 out of 5 (59%) vs. 7.6 ± 2.5 out of 14 (54%)), so we could conclude that they showed good general knowledge on how a MedD menu should be made.

If we go back to the turn of the 19th to 20th century, mainland Croatia’s diet was based mostly on stews, soups, pork and its processed meat, poultry, dairy products, and cakes. Coastal Croatia’s diet was based mostly on stews, a lot of vegetables, olive oil, fish, dairy products, and occasionally meat [79]. These traditional dietary patterns became less pronounced in younger generations [59,64,71,80,81,82,83,84], which was also observed in our study. Interestingly, parents’ diet also has a high impact on children’s diet quality [85], which is key when advancing from planned kindergarten menus to primary schools where not all children follow the nutritionists’-tailored menus [65]. Therefore, particular groups, such as adolescents, students, and pregnant women, as well as men, should be considered when planning further awareness raising and educational campaigns, which should also be focused on the financial aspects of diet planning.

Based on our logistic regression analysis, we found that sex, residence, and income were major predictors for MedD score. Assuming that a person would like to keep her/his area of residence, and that sex is rather unchangeable, we wanted to target the low-income population for refinement of their dietary plan based on the MedD principles. Our calculations actually showed that the MedD plan is on average slightly less expensive compared to the healthy omnivore plan; however, we did not compare it to unbalanced dietary plans that are often the reason for weight gain. Moreover, using the dietary plan tailoring, it is possible to eat according to MedD principles on a budget. Our calculations have the limitation of choosing prices from a nation-wide retailer, and some costs might be lower or higher in some other shops or during special offers. A major obstacle in dietary planning is the seasonality of fruit and vegetables, and although they are now available the whole year long, their price varies considerably in the winter period. Still, with a slight change in meat protein intake (pork instead of veal or mussels instead of prawns) and choosing different fruit (pears, oranges, kiwi, etc.) and vegetables (cabbage, broccoli, etc.), it is possible to make corrections within the plan, thus not altering the budget. Previous meta-analysis found that healthier food options costed slightly more (USD 0.29 per serving), and that the top quantile compared to the low quantile costed USD 1.54 per 2000 kcal more [86]. As a possible solution on how to tackle this issue, application of higher tax rates for “unhealthy” food items would contribute to price balancing, and potentially lead to substantial savings in healthcare system costs by preventing many NCDs [87]. Although nutrient-dense food is more expensive per calorie, recent findings [88,89,90] suggest that, with proper planning, it is possible to combine food items that are nutrient rich, affordable, and culturally acceptable. It is therefore necessary to initiate research that would tackle the assumptions that a “healthy” diet means higher expenses, and data presented in Figure 7 goes in line with such recommendations.

## 5. Conclusions

To conclude, in this study we showed that the Croatian population moderately practices adherence to Mediterranean diet. We also identified that more focus in promoting healthy aspects of MedD should be made towards men, people residing in the continental part of Croatia, adolescents and young adults, as well as toward people with incomes below the national median. Based on the available literature, the situation in Croatia reflects trends in other Mediterranean countries, as well as “Western countries”, so similar approaches to improve nutrition quality could be implemented. By turning to MedD and improving lifestyle, it is possible to save money, prevent diseases, and even navigate ageing [91]; are not these enough to take action?

## Data Availability

The original contributions generated for this study are included in the article. Further inquiries can be directed to the corresponding author upon reasonable request.

## References

[1] WHO (2020). Global Health Estimates 2020: Deaths by Cause, Age, Sex, by Country and by Region, 2000–2019.

[2] Ritchie H., Roser M. Causes of Death—Our World in Data. https://ourworldindata.org/causes-of-death#citation.

[3] Poirier P., Giles T.D., Bray G.A., Hong Y., Stern J.S., Pi-Sunyer F.X., Eckel R.H. (2006). Obesity and Cardiovascular Disease: Pathophysiology, Evaluation, and Effect of Weight Loss: An Update of the 1997 American Heart Association Scientific Statement on Obesity and Heart Disease from the Obesity Committee of the Council on Nutrition, Physical. Circulation.

[4] D’Agostino R.B., Vasan R.S., Pencina M.J., Wolf P.A., Cobain M., Massaro J.M., Kannel W.B. (2008). General Cardiovascular Risk Profile for Use in Primary Care: The Framingham Heart Study. Circulation.

[5] Lim S.S., Vos T., Flaxman A.D., Danaei G., Shibuya K., Adair-Rohani H., Amann M., Anderson H.R., Andrews K.G., Aryee M. (2012). A Comparative Risk Assessment of Burden of Disease and Injury Attributable to 67 Risk Factors and Risk Factor Clusters in 21 Regions, 1990-2010: A Systematic Analysis for the Global Burden of Disease Study 2010. Lancet.

[6] Achilleos S., Quattrocchi A., Gabel J., Heraclides A., Kolokotroni O., Constantinou C., Ugarte M.P., Nicolaou N., Rodriguez-Llanes J.M., Bennett C.M. (2022). Excess All-Cause Mortality and COVID-19-Related Mortality: A Temporal Analysis in 22 Countries, from January until August 2020. Int. J. Epidemiol..

[7] Williamson E.J., Walker A.J., Bhaskaran K., Bacon S., Bates C., Morton C.E., Curtis H.J., Mehrkar A., Evans D., Inglesby P. (2020). Factors Associated with COVID-19-Related Death Using OpenSAFELY. Nature.

[8] Richardson S., Hirsch J.S., Narasimhan M., Crawford J.M., McGinn T., Davidson K.W., Barnaby D.P., Becker L.B., Chelico J.D., Cohen S.L. (2020). Presenting Characteristics, Comorbidities, and Outcomes among 5700 Patients Hospitalized with COVID-19 in the New York City Area. JAMA.

[9] (2021). EUROSTAT Overweight and Obesity—BMI Statistics—Statistics Explained. https://ec.europa.eu/eurostat/statistics-explained/index.php?title=Overweight_and_obesity_-_BMI_statistics.

[10] Di Cesare M., Bentham J., Stevens G.A., Zhou B., Danaei G., Lu Y., Bixby H., Cowan M.J., Riley L.M., Hajifathalian K. (2016). Trends in Adult Body-Mass Index in 200 Countries from 1975 to 2014: A Pooled Analysis of 1698 Population-Based Measurement Studies with 19.2 Million Participants. Lancet.

[11] Milanović S.M., Morović M.L., Bukal D., Križan H., Buoncristiano M., Breda J. (2020). Regional and Sociodemographic Determinants of the Prevalence of Overweight and Obesity in Children Aged 7-9 Years in Croatia. Acta Clin. Croat..

[12] Rito A.I., Buoncristiano M., Spinelli A., Salanave B., Kunešová M., Hejgaard T., Solano M.G., Fijałkowska A., Sturua L., Hyska J. (2019). Association between Characteristics at Birth, Breastfeeding and Obesity in 22 Countries: The WHO European Childhood Obesity Surveillance Initiative—COSI 2015/2017. Obes. Facts.

[13] Spinelli A., Buoncristiano M., Nardone P., Starc G., Hejgaard T., Júlíusson P.B., Fismen A.S., Weghuber D., Musić Milanović S., García-Solano M. (2021). Thinness, Overweight, and Obesity in 6- to 9-Year-Old Children from 36 Countries: The World Health Organization European Childhood Obesity Surveillance Initiative—COSI 2015–2017. Obes. Rev..

[14] Seidell J.C., Halberstadt J. (2015). The Global Burden of Obesity and the Challenges of Prevention. Ann. Nutr. Metab..

[15] Haslam D.W., James W.P.T. (2005). Obesity. Lancet.

[16] Viegas S., Ladeira C., Costa-veiga A., Perelman J., Gajski G. (2017). Forgotten Public Health Impacts of Cancer—An Overview. Arh Hig. Rada Toksikol..

[17] European Commission (2020). Special Eurobaromter 505—Making our Food Fit for the Future—Citizens’ Expectations.

[18] Andreyeva T., Long M.W., Brownell K.D. (2010). The impact of food prices on consumption: A systematic review of research on the price elasticity of demand for food. Am. J. Public Health.

[19] Estruch R., Ros E., Salas-Salvadó J., Covas M.-I., Corella D., Arós F., Gómez-Gracia E., Ruiz-Gutiérrez V., Fiol M., Lapetra J. (2018). Primary Prevention of Cardiovascular Disease with a Mediterranean Diet Supplemented with Extra-Virgin Olive Oil or Nuts. N. Engl. J. Med..

[20] Dernini S., Berry E.M., Serra-Majem L., La Vecchia C., Capone R., Medina F.X., Aranceta-Bartrina J., Belahsen R., Burlingame B., Calabrese G. (2017). Med Diet 4.0: The Mediterranean Diet with Four Sustainable Benefits. Public Health Nutr..

[21] Molina-Montes E., Ubago-Guisado E., Petrova D., Amiano P., Chirlaque M.D., Agudo A., Sánchez M.J. (2021). The Role of Diet, Alcohol, Bmi, and Physical Activity in Cancer Mortality: Summary Findings of the Epic Study. Nutrients.

[22] Sofi F., Abbate R., Gensini G.F., Casini A. (2010). Accruing Evidence on Benefits of Adherence to the Mediterranean Diet on Health: An Updated Systematic Review and Meta-Analysis. Am. J. Clin. Nutr..

[23] De Filippis F., Pellegrini N., Vannini L., Jeffery I.B., La Storia A., Laghi L., Serrazanetti D., Di Cagno R., Ferrocino I., Lazzi C. (2016). High-Level Adherence to a Mediterranean Diet Beneficially Impacts the Gut Microbiota and Associated Metabolome. Gut.

[24] D’Alessandro A., Lampignano L., De Pergola G. (2019). Mediterranean Diet Pyramid: A Proposal for Italian People. A Systematic Review of Prospective Studies to Derive Serving Sizes. Nutrients.

[25] Bach-Faig A., Berry E.M., Lairon D., Reguant J., Trichopoulou A., Dernini S., Medina F.X., Battino M., Belahsen R., Miranda G. (2011). Mediterranean Diet Pyramid Today Science and Cultural Updates. Public Health Nutr..

[26] Davis C., Bryan J., Hodgson J., Murphy K. (2015). Definition of the Mediterranean Diet; a Literature Review. Nutrients.

[27] Vilarnau C., Stracker D.M., Funtikov A., da Silva R., Estruch R., Bach-Faig A. (2019). Worldwide Adherence to Mediterranean Diet between 1960 and 2011. Eur. J. Clin. Nutr..

[28] Kolčić I., Relja A., Gelemanović A., Miljković A., Boban K., Hayward C., Rudan I., Polašek O. (2016). Mediterranean Diet in the Southern Croatia—Does It Still Exist?. Croat. Med. J..

[29] Schröder H., Fitó M., Estruch R., Martínez-González M.A., Corella D., Salas-Salvadó J., Lamuela-Raventós R., Ros E., Salaverría I., Fiol M. (2011). A Short Screener Is Valid for Assessing Mediterranean Diet Adherence among Older Spanish Men and Women. J. Nutr..

[30] EFSA (2021). Food Consumption Data.

[31] Ocké M., Boer E., De Brants H., Laan J. (2012). External Efsa Scientific Report: Pancake—Pilot Study for the Assessment of Nutrient Intake and Food Consumption Among Kids in Europe. EFSA.

[32] U.S. Department of Agriculture (2020). U.S. Department of Health and Human Services Dietary Guidelines for Americans 2020–2025.

[33] Oldways: A Food And Nutrition Nonprofit Helping People Live Healthier, Happier Lives. https://oldwayspt.org/traditional-diets/mediterranean-diet/traditional-med-diet.

[34] van Buuren S., Groothuis-Oudshoorn K. (2011). Mice: Multivariate Imputation by Chained Equations in R. J. Stat. Softw..

[35] Bentham J., Di Cesare M., Bilano V., Bixby H., Zhou B., Stevens G.A., Riley L.M., Taddei C., Hajifathalian K., Lu Y. (2017). Worldwide Trends in Body-Mass Index, Underweight, Overweight, and Obesity from 1975 to 2016: A Pooled Analysis of 2416 Population-Based Measurement Studies in 128·9 Million Children, Adolescents, and Adults. Lancet.

[36] Afshin A., the GBD 2015 Obesity Collaborators (2017). Health Effects of Overweight and Obesity in 195 Countries over 25 Years. N. Engl. J. Med..

[37] Iacobini C., Vitale M., Haxhi J., Pesce C., Pugliese G., Menini S. (2022). Food-Related Carbonyl Stress in Cardiometabolic and Cancer Risk Linked to Unhealthy Modern Diet. Nutrients.

[38] Rubio-Tomás T., Rueda-Robles A., Plaza-Díaz J., Álvarez-Mercado A.I. (2022). Nutrition and cellular senescence in obesity-related disorders. J. Nutr. Biochem..

[39] Kopelman P.G. (2000). Obesity as a medical problem. Nature.

[40] Sánchez M.G., Sánchez L.G., Patino-Alonso M.C., Alonso-Domínguez R., Sánchez-Aguadero N., Lugones-Sánchez C., Sánchez E.R., Ortiz L.G., Gómez-Marcos M.A. (2020). Adherence to the Mediterranean Diet in Spanish Population and Its Relationship with Early Vascular Aging According to Sex and Age: EVA Study. Nutrients.

[41] Jones J.P.H., Abdullah M.M.H., Wood D., Jones P.J.H. (2019). Economic Modeling for Improved Prediction of Saving Estimates in Healthcare Costs from Consumption of Healthy Foods: The Mediterranean-Style Diet Case Study. Food Nutr. Res..

[42] Sofi F., Macchi C., Abbate R., Gensini G.F., Casini A. (2014). Mediterranean Diet and Health Status: An Updated Meta-Analysis and a Proposal for a Literature-Based Adherence Score. Public Health Nutr..

[43] Tognon G., Lissner L., Sæbye D., Walker K.Z., Heitmann B.L. (2014). The Mediterranean Diet in Relation to Mortality and CVD: A Danish Cohort Study. Br. J. Nutr..

[44] Trichopoulou A., Costacou T., Bamia C., Trichopoulos D. (2009). Adherence to a Mediterranean Diet and Survival in a Greek Population. Vasc. Med..

[45] Castelló A., Boldo E., Pérez-Gómez B., Lope V., Altzibar J.M., Martín V., Castaño-Vinyals G., Guevara M., Dierssen-Sotos T., Tardón A. (2017). Adherence to the Western, Prudent and Mediterranean Dietary Patterns and Breast Cancer Risk: MCC-Spain Study. Maturitas.

[46] Sureda A., del Mar Bibiloni M., Julibert A., Bouzas C., Argelich E., Llompart I., Pons A., Tur J.A. (2018). Adherence to the Mediterranean Diet and Inflammatory Markers. Nutrients.

[47] Lagiou P., Trichopoulos D., Sandin S., Lagiou A., Mucci L., Wolk A., Weiderpass E., Adami H.-O. (2006). Mediterranean Dietary Pattern and Mortality among Young Women: A Cohort Study in Sweden. Br. J. Nutr..

[48] Gajski G., Gerić M., Vučić Lovrenčić M., Božičević S., Rubelj I., Nanić L., Škrobot Vidaček N., Bendix L., Peraica M., Rašić D. (2018). Analysis of Health-Related Biomarkers between Vegetarians and Non-Vegetarians: A Multi-Biomarker Approach. J. Funct. Foods.

[49] Gajski G., Gerić M., Jakaša I., Peremin I., Domijan A.M., Vučić Lovrenčić M., Kežić S., Bituh M., Moraes de Andrade V. (2021). Inflammatory, Oxidative and DNA Damage Status in Vegetarians: Is the Future of Human Diet Green?. Crit. Rev. Food Sci. Nutr..

[50] Scoditti E., Tumolo M.R., Garbarino S. (2022). Mediterranean Diet on Sleep: A Health Alliance. Nutrients.

[51] Evans J.R., Mathur A. (2018). The Value of Online Surveys: A Look Back and a Look Ahead. Internet Res..

[52] Smith W.G. (2008). Does Gender Influence Online Survey Participation? A Record-Linkage Analysis of University Faculty Online Survey Response Behavior.

[53] Havaš Auguštin D., Šarac J., Lovrić M., Živković J., Malev O., Fuchs N., Novokmet N., Turkalj M., Missoni S. (2020). Adherence to Mediterranean Diet and Maternal Lifestyle during Pregnancy: Island–Mainland Differentiation in the CRIBS Birth Cohort. Nutrients.

[54] Dinu M., Pagliai G., Giangrandi I., Colombini B., Toniolo L., Gensini G., Sofi F. (2021). Adherence to the Mediterranean Diet among Italian Adults: Results from the Web-Based Medi-Lite Questionnaire. Int. J. Food Sci. Nutr..

[55] Pfeifer D., Rešetar J., Gajdoš Kljusurić J., Panjkota Krbavčić I., Vranešić Bender D., Rodríguez-Pérez C., Ruíz-López M.D., Šatalić Z. (2021). Cooking at Home and Adherence to the Mediterranean Diet During the COVID-19 Confinement: The Experience From the Croatian COVIDiet Study. Front. Nutr..

[56] Cuschieri S., Libra M. (2021). Adherence to the Mediterranean Diet in Maltese Adults. Int. J. Environ. Res. Public Health.

[57] del Mar Bibiloni M., González M., Julibert A., Llompart I., Pons A., Tur J.A. (2017). Ten-Year Trends (1999–2010) of Adherence to the Mediterranean Diet among the Balearic Islands’ Adult Population. Nutrients.

[58] Crichton G.E., Bryan J., Hodgson J.M., Murphy K.J. (2013). Mediterranean Diet Adherence and Self-Reported Psychological Functioning in an Australian Sample. Appetite.

[59] Fiore M., Ledda C., Rapisarda V., Sentina E., Mauceri C., DAgati P., Conti G.O., Serra-Majem L., Ferrante M. (2015). Medical School Fails to Improve Mediterranean Diet Adherence among Medical Students. Eur. J. Public Health.

[60] Grosso G., Marventano S., Buscemi S., Scuderi A., Matalone M., Platania A., Giorgianni G., Rametta S., Nolfo F., Galvano F. (2013). Factors Associated with Adherence to the Mediterranean Diet among Adolescents Living in Sicily, Southern Italy. Nutrients.

[61] Grosso G., Marventano S., Giorgianni G., Raciti T., Galvano F., Mistretta A. (2014). Mediterranean Diet Adherence Rates in Sicily, Southern Italy. Public Health Nutr..

[62] Papadaki A., Wood L., Sebire S.J., Jago R. (2015). Adherence to the Mediterranean Diet among Employees in South West England: Formative Research to Inform a Web-Based, Work-Place Nutrition Intervention. Prev. Med. Rep..

[63] Pelucchi C., Galone C., Negri E., La Vecchia C. (2010). Trends in Adherence to the Mediterranean Diet in an Italian Population between 1991 and 2006. Eur. J. Clin. Nutr..

[64] Ruggiero E., Di Castelnuovo A., Costanzo S., Persichillo M., Bracone F., Cerletti C., Donati M.B., De Gaetano G., Iacoviello L., Bonaccio M. (2019). Socioeconomic and Psychosocial Determinants of Adherence to the Mediterranean Diet in a General Adult Italian Population. Eur. J. Public Health.

[65] Obradovic Salcin L., Karin Z., Damjanovic V.M., Ostojic M., Vrdoljak A., Gilic B., Sekulic D., Lang-Morovic M., Markic J., Sajber D. (2019). Physical Activity, Body Mass, and Adherence to the Mediterranean Diet in Preschool Children: A Cross-Sectional Analysis in the Split-Dalmatia County (Croatia). Int. J. Environ. Res. Public Health.

[66] Pavičić Žeželj S., Jovanović G.K., Zubalj N.D., Mićović V., Sesar Ž. (2018). Associations between Adherence to the Mediterranean Diet and Lifestyle Assessed with the MEDLIFE Index among the Working Population. Int. J. Environ. Res. Public Health.

[67] Štefan L., Čule M., Milinović I., Sporiš G., Juranko D. (2017). The Relationship between Adherence to the Mediterranean Diet and Body Composition in Croatian University Students. Eur. J. Integr. Med..

[68] Aljabri M.K., Al-Raddadi R., Bahijri S.M., Al Ahmadi J., Ajabnoor G., Jambi H.A. (2019). Factors Associated with Adherence to Mediterranean Diet among Saudi Non-Diabetic Patients Attending Primary Health Care Centers: A Cross-Sectional Study. J. Taibah Univ. Med. Sci..

[69] Bottcher M.R., Marincic P.Z., Nahay K.L., Baerlocher B.E., Willis A.W., Park J., Gaillard P., Greene M.W. (2017). Nutrition Knowledge and Mediterranean Diet Adherence in the Southeast United States: Validation of a Field-Based Survey Instrument. Appetite.

[70] Rodrigues S., Caraher M., Trichopoulou A., de Almeida M. (2008). Portuguese Households’ Diet Quality (Adherence to Mediterranean Food Pattern and Compliance with WHO Population Dietary Goals): Trends, Regional Disparities and Socioeconomic Determinants. Eur. J. Clin. Nutr..

[71] Baydemir C., Ozgur E.G., Balci S. (2018). Evaluation of Adherence to Mediterranean Diet in Medical Students at Kocaeli University, Turkey. J. Int. Med. Res..

[72] Robine J.M., Herrmann F.R., Arai Y., Willcox D.C., Gondo Y., Hirose N., Suzuki M., Saito Y. (2013). Accuracy of the Centenarian Numbers in Okinawa and the Role of the Okinawan Diet on Longevity. Responses to Le Bourg about the Article “Exploring the Impact of Climate on Human Longevity. Exp. Gerontol..

[73] Willcox D., John Willcox B., Yasura S., Willcox D., Willcox B., Yasura S., Ashitomi I., Suzuki M. (2012). Gender Gap in Healthspan and Life Expectancy in Okinawa: Health Behaviours. Asian J. Gerontol. Geriatr..

[74] Willcox B.J., Willcox D.C., Todoriki H., Fujiyoshi A., Yano K., He Q., Curb J.D., Suzuki M. (2007). Caloric Restriction, the Traditional Okinawan Diet, and Healthy Aging: The Diet of the World’s Longest-Lived People and Its Potential Impact on Morbidity and Life Span. Ann. N. Y. Acad. Sci..

[75] Ferrières J. (2004). The French Paradox: Lessons for Other Countries. Heart.

[76] Abdelhamid A.S., Brown T.J., Brainard J.S., Biswas P., Thorpe G.C., Moore H.J., Deane K.H.O., Summerbell C.D., Worthington H.V., Song F. (2020). Omega-3 Fatty Acids for the Primary and Secondary Prevention of Cardiovascular Disease. Cochrane Database Syst. Rev..

[77] George E.S., Marshall S., Mayr H.L., Trakman G.L., Tatucu-Babet O.A., Lassemillante A.C.M., Bramley A., Reddy A.J., Forsyth A., Tierney A.C. (2019). The Effect of High-Polyphenol Extra Virgin Olive Oil on Cardiovascular Risk Factors: A Systematic Review and Meta-Analysis. Crit. Rev. Food Sci. Nutr..

[78] Schwingshackl L., Hoffmann G. (2014). Monounsaturated Fatty Acids, Olive Oil and Health Status: A Systematic Review and Meta-Analysis of Cohort Studies. Lipids Health Dis..

[79] Briški M., Jarec M. (2014). Cultural and Geographical Overview of Traditional Diet in Croatia. Coll. Antropol..

[80] Šarac J., Auguštin D.H., Lovrić M., Stryeck S., Šunić I., Novokmet N., Missoni S. (2021). A Generation Shift in Mediterranean Diet Adherence and Its Association with Biological Markers and Health in Dalmatia, Croatia. Nutrients.

[81] Colić-Barić I., Kajfež R., Šatalić Z., Cvjetić S. (2004). Comparison of Dietary Habits in the Urban and Rural Croatian Schoolchildren. Eur. J. Nutr..

[82] Sarić M.M., Ljubičić M., Lapčić I., Guiné R.P.F. (2020). Contribution of Fruit, Vegetables, Whole Cereals, and Legumes to Total Fibre Intake in Adult Croatian Dalmatian Population. Arh. Hig. Rada Toksikol..

[83] Ljubičić M., Sarić M.M., Barić I.C., Rumbak I., Komes D., Šatalić Z., Guiné R.P.F. (2017). Consumer Knowledge and Attitudes toward Healthy Eating in Croatia: A Cross-Sectional Study. Arh. Hig. Rada Toksikol..

[84] Ilić A., Rumbak I., Marić L., Karlović T., Brečić R., Colić Barić I., Bituh M. (2019). The Proportion of Differently Processed Foods in the Diet of Croatian School-Aged Children and Its Impact on Daily Energy and Nutrient Intake. Croat. J. Food Sci. Technol..

[85] Krešić G., Kenđel Jovanović G., Pavičić Žeželj S., Pleadin J., Liović N., Plepel K. (2018). Parental Adherence to Mediterranean Diet Is Associated with Their Adolescents’ Cereals Intake. Croat. J. Food Sci. Technol..

[86] Rao M., Afshin A., Singh G., Mozaffarian D. (2013). Do Healthier Foods and Diet Patterns Cost More than Less Healthy Options? A Systematic Review and Meta-Analysis. BMJ Open.

[87] Mozaffarian D., Afshin A., Benowitz N.L., Bittner V., Daniels S.R., Franch H.A., Jacobs D.R., Kraus W.E., Kris-Etherton P.M., Krummel D.A. (2012). Population Approaches to Improve Diet, Physical Activity, and Smoking Habits: A Scientific Statement from the American Heart Association. Circulation.

[88] Lee A.J., Kane S., Ramsey R., Good E., Dick M. (2016). Testing the Price and Affordability of Healthy and Current (Unhealthy) Diets and the Potential Impacts of Policy Change in Australia. BMC Public Health.

[89] Drewnowski A., Eichelsdoerfer P. (2009). The Mediterranean Diet: Does It Have to Cost More?. Public Health Nutr..

[90] Darmon N., Drewnowski A. (2015). Contribution of food prices and diet cost to socioeconomic disparities in diet quality and health: A systematic review and analysis. Nutr. Rev..

[91] Vidaček N.Š., Nanić L., Ravlić S., Sopta M., Gerić M., Gajski G., Garaj-Vrhovac V., Rubelj I. (2018). Telomeres, Nutrition, and Longevity: Can We Really Navigate Our Aging?. J. Gerontol. Ser. A Biol. Sci. Med. Sci..

